# Indirect measurement of anterior-posterior ground reaction forces using a minimal set of wearable inertial sensors: from healthy to hemiparetic walking

**DOI:** 10.1186/s12984-020-00700-7

**Published:** 2020-06-29

**Authors:** Dheepak Arumukhom Revi, Andre M. Alvarez, Conor J. Walsh, Stefano M.M. De Rossi, Louis N. Awad

**Affiliations:** 1 College of Health and Rehabilitation Sciences, Boston University, Boston, Massachusetts, USA; 2 Wyss Institute for Biologically Inspired Engineering, Harvard University, Cambridge, Massachusetts, USA; 3 John A. Paulson School of Engineering and Applied Science, Harvard University, Cambridge, Massachusetts, USA

**Keywords:** Ground reaction forces, Estimation, Wearable sensors, Walking, Propulsion, Hemiparetic

## Abstract

**Background:**

The anterior-posterior ground reaction force (AP-GRF) and propulsion and braking point metrics derived from the AP-GRF time series are indicators of locomotor function across healthy and neurological diagnostic groups. In this paper, we describe the use of a minimal set of wearable inertial measurement units (IMUs) to indirectly measure the AP-GRFs generated during healthy and hemiparetic walking.

**Methods:**

Ten healthy individuals and five individuals with chronic post-stroke hemiparesis completed a 6-minute walk test over a walking track instrumented with six forceplates while wearing three IMUs securely attached to the pelvis, thigh, and shank. Subject-specific models driven by IMU-measured thigh and shank angles and an estimate of body acceleration provided by the pelvis IMU were used to generate indirect estimates of the AP-GRF time series. Propulsion and braking point metrics (i.e., peaks, peak timings, and impulses) were extracted from the IMU-generated time series. Peaks and impulses were expressed as % bodyweight (%bw) and peak timing was expressed as % stance phase (%sp). A 75%-25% split of 6-minute walk test data was used to train and validate the models. Indirect estimates of the AP-GRF time series and point metrics were compared to direct measurements made by the forceplates.

**Results:**

Indirect measurements of the AP-GRF time series approximated the direct measurements made by forceplates, with low error and high consistency in both the healthy (*RMSE*= 4.5%bw; *R*^2^= 0.93) and post-stroke (*RMSE*= 2.64%bw; *R*^2^= 0.90) cohorts. In the healthy cohort, the average errors between indirect and direct measurements of the peak propulsion magnitude, peak propulsion timing, and propulsion impulse point estimates were 2.37%bw, 0.67%sp, and 0.43%bw. In the post-stroke cohort, the average errors for these point estimates were 1.07%bw, 1.27%sp, and 0.31%bw. Average errors for the braking estimates were higher, but comparable.

**Conclusions:**

Accurate estimates of AP-GRF metrics can be generated using three strategically mounted IMUs and subject-specific calibrations. This study advances the development of point-of-care diagnostic systems that can catalyze the routine assessment and management of propulsion and braking locomotor deficits during rehabilitation.

The neuromechanical processes underlying healthy bipedal locomotion are multi-factorial [1–3] and converge on locomotor patterns that are characteristically fast, efficient, and stable [1, 4]. An impaired ability to transition from step to step is a locomotor deficit common across diagnostic groups [5–13]. During the step-to-step transition of each gait cycle, a braking force is generated by the leading limb as it makes contact with the ground in front of the body. To efficiently accelerate the body into the next step, coordination of the timing and magnitude of the forward propulsion force generated by the trailing limb is required [1, 14–16]. Moreover, to walk faster, healthy individuals symmetrically increase the magnitude of propulsion generated by each limb while maintaining the relative timing of the propulsion peak [15, 17, 18]. In individuals with impaired propulsion function, walking is often slow, metabolically expensive, and unstable [19–22].

Laboratory equipment such as instrumented treadmills and forceplates are the gold standard in characterizing propulsion and braking function during healthy [23, 24] and impaired [5, 6, 9, 10, 20, 25–27] walking by way of direct measurements of the anterior-posterior ground reaction forces (AP-GRFs) generated during walking and point metrics extracted from the AP-GRF time series (Fig. 1). For example, older adults are reported to generate up to 22% less peak propulsion (i.e., the peak of the anterior ground reaction force) compared to young adults [23, 24], and in people post-stroke, the propulsion generated by the paretic limb is up to 68% less than the non-paretic limb [9, 20, 26, 27]. Studies that have combined AP-GRF measurements with clinical evaluations have shown the clinical consequences of impaired propulsion function. Indeed, asymmetry in the propulsion impulses generated by the paretic and non-paretic limbs is correlated with hemiparetic severity [9, 28]. Moreover, deficits in propulsion function are highly related to walking speed [29] and long distance walking [30] after stroke—key determinants of community participation and perceived quality of life [19, 31, 32].

**Fig. 1:**
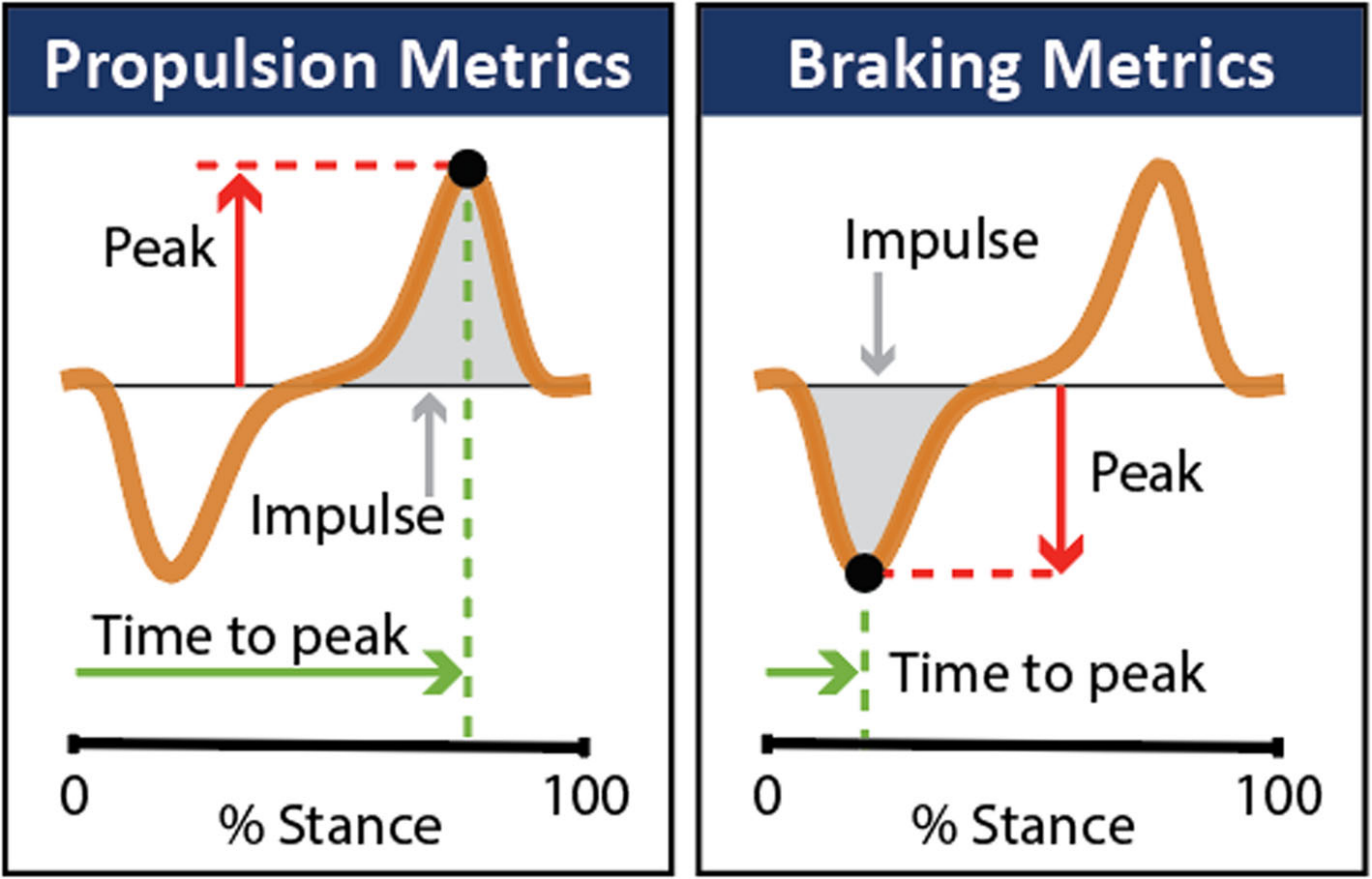
Anterior-posterior ground reaction force (AP-GRF) time series and salient propulsion and braking metrics

Despite the importance of propulsion to a functional bipedal gait, conventional rehabilitation efforts have, by and large, been unable to restore propulsion function after neurological injury or dysfunction. The development and study of interventions that target propulsion function is a highly active area of research [12, 33–41]; however, the clinical translation of these experimental approaches is hindered by the limited access that rehabilitation clinicians have to the sophisticated instrumentation (i.e., forceplates and instrumented treadmills) and personnel with advanced training required to collect, analyze, and interpret ground reaction force data. Moreover, even in settings with access to a motion analysis laboratory, locomotor differences inherent to treadmill walking and the small collection footprint of most overground forceplate walkways limit ecological validity. Together, these limitations of the current state-of-the-art motivate the development of point-of-care propulsion diagnostic systems. The clinical management of locomotor propulsion deficits will remain untenable if the measurement instruments used to assess limb propulsion remain inaccessible.

Wearable sensors are a promising solution for this measurement gap. Indeed, wearable sensors have been used to extend gait measurements outside of the laboratory [42–47] and a wide range of methods and sensors have proven effective in providing indirect measurements of the ground reaction forces generated during walking [48–51]. These methods, however, have largely not been effective for the AP-GRFs and depend on assumptions of healthy, consistent walking patterns that may not translate to impaired locomotor patterns [51, 52]. Recent work has shown that inertial measurement units (IMUs) can be used to make measurements during healthy [44] and hemiparetic walking [53] that are highly correlated to key features of propulsion. The aims of this study were to extend this work by describing the use of a minimal set of IMUs to indirectly measure the AP-GRF generated during healthy and hemiparetic walking and provide estimates of: (i) the AP-GRF time series and (ii) salient propulsion and braking point metrics (i.e., peak magnitudes, peak timings, and impulses) extracted from the time series (see Fig. 1).

## Methods

### Participants

Ten healthy individuals (26±4 years, 171±10.8 cm, 68±17 kg) (Table 1, top) who were free of conditions that impaired walking ability (as per self-report and confirmation during the study visit) and five individuals with chronic post-stroke hemiparesis (58±15 years, 180±2.9 cm, 91±12 kg) (Table 1, bottom) were recruited to participate in this study. The inclusion criteria for study participants who were post-stroke consisted of being greater than six months post-stroke, having the ability to walk without the assistance of another individual, and presenting with observable gait deficits. Exclusion criteria included comorbidities other than stroke that impaired walking ability, resting heart rate outside the range of 40 to 100 beats per minute, resting blood pressure outside the range of 90/60 to 170/90 mmHg, inability to communicate with investigators, and pain in the lower limbs or spine. Individuals post-stroke were recruited from a research participant registry generated from the clinical programs at Boston University, referrals from local clinics and hospitals, and flyers distributed in and around Boston. All study procedures were approved by the Boston University Institutional Review Board and written informed consent was secured from all study participants.

**Table 1:** Study participant characteristics

**Participant number**	**Side of Paresis**	**Stroke onset (y)**	**Sex**	**Age (y)**	**Height (cm)**	**Weight (kg)**	**CWS (m/s)**	**Pp (%)**
*Healthy study participants*								
H01	-	-	F	33	155	54.0	1.10	-
H02	-	-	F	25	164	46.9	1.29	-
H03	-	-	F	24	174	64.6	1.64	-
H04	-	-	M	29	179	63.9	1.19	-
H05	-	-	F	23	154	55.6	1.55	-
H06	-	-	F	25	162	64.4	1.36	-
H07	-	-	M	21	179	57.6	1.25	-
H08	-	-	M	27	179	101.2	1.22	-
H09	-	-	M	30	177	91.2	1.23	-
H10	-	-	M	25	183	78.0	1.42	-
Average ±SD	-	-	-	26 ±4	171 ±11	68 ±17	1.33 ±0.17	-
*Study participants with post-stroke hemiparesis*								
S01	Left	8.08	M	61	180	72.6	0.97	18
S02	Right	5.92	M	35	184	93.0	1.47	31
S03	Left	7.92	M	78	181	100.8	1.00	32
S04	Right	7.25	M	56	180	88.0	0.80	24
S05	Left	6.08	M	62	176	99.8	1.27	52
Average ±SD	-	7.1 ±1.0	-	58 ±15	180 ±2.9	91 ±12	1.10 ±0.27	31 ±13

Abbreviations: CWS - comfortable walking speed, Pp - propulsion symmetry (see [9])

### Gait evaluation and data collection overview

**Fig. 2:**
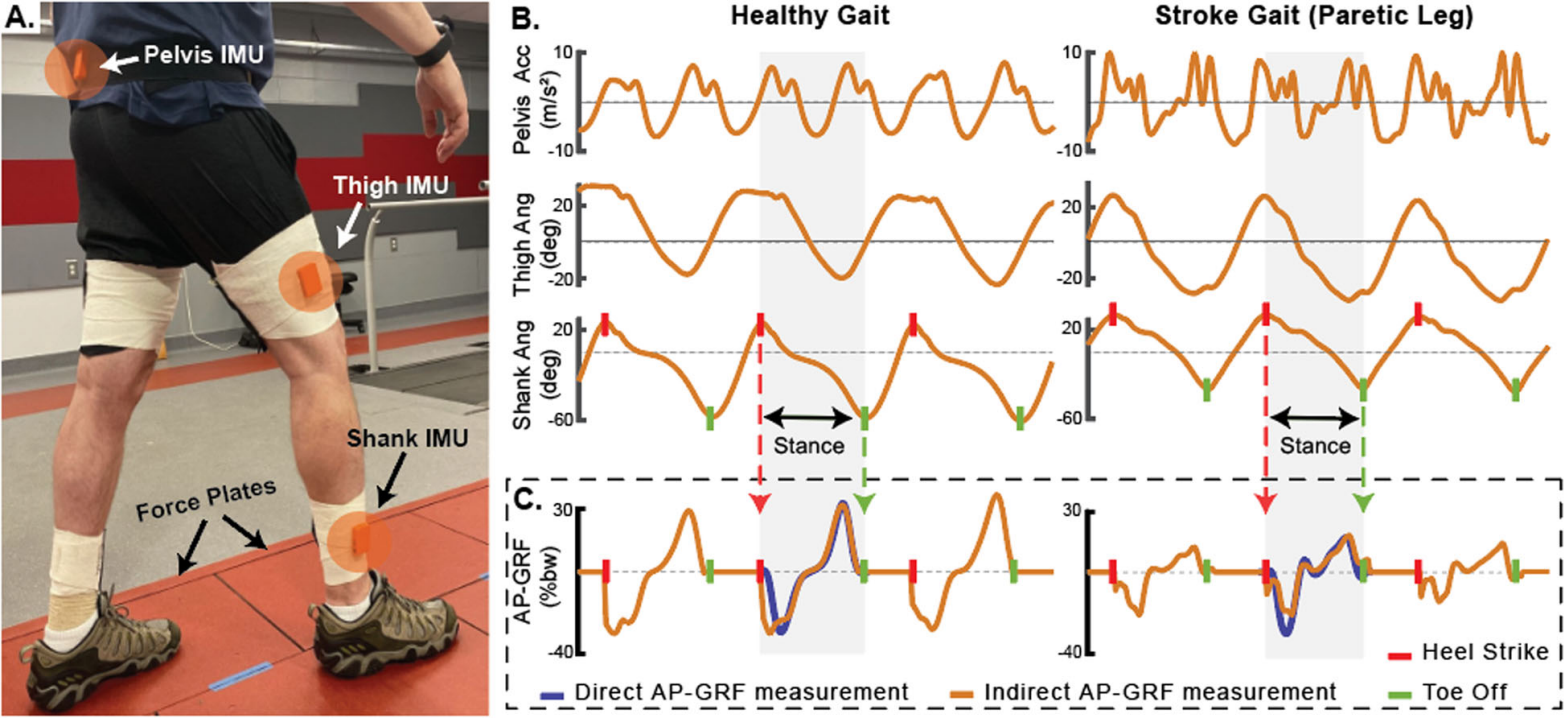
**a** IMU setup on a participant and walking track with forceplates mounted level with the surrounding floor. **b** IMU-measured pelvis acceleration and shank and thigh angles were used in combination with subject-specific estimation algorithms to generate indirect measurements of the anterior-posterior ground reaction force (AP-GRF). Moreover, the shank angle was used to identify the stance phase, defined as the period between heel strike and toe off of the target limb. **c** IMU-based AP-GRF (indirect) and forceplate measured AP-GRF (direct). Direct AP-GRF measurements were limited to forceplate strikes, thus one stride

Study participants completed a testing session that included a standing static trial, 10-meter walk test at a comfortable walking speed (CWS), and a 6-minute walk test with the instruction to cover as much distance as safely possible [54]. The standing static trial served as a reference for the orientation of the IMUs, the 10-meter walk test was used to measure usual walking speed and paretic propulsion symmetry (as described in [9]) to characterize study participants’ baseline walking function (see Table 1), and the 6-minute walk test provided the model training and validation datasets. All walking tests were performed around a 26.6m oval indoor track consisting of two 10m straightaways separated by 3.3m turns on either end. One of the 10m straightaways was instrumented with six forceplates (Bertec, Columbus, OH, USA) located level with the surrounding floor (Fig. 2a) to enable the collection of ground reaction forces (i.e., the reference standard) during the 6-minute walk test.

Prior to testing, wireless inertial measurement units (IMUs, MTw Awinda, Xsens, Enschede, Netherlands) were securely attached to the posterior pelvis and laterally on the thigh and shank using fabric wraps (Fig. 2a). These three IMUs served as the primary sensor set evaluated in this study, with each IMU selected based on a biomechanics-based model of the propulsion and braking forces generated during walking. More specifically, when considered together, the shank and thigh sagittal angles measured by the IMUs attached to these segments provide information on the limb’s position relative to the body [44] and the acceleration measured by the pelvis IMU serves as a proxy for the body acceleration. Both the limb’s position relative to the body and the body’s acceleration during walking are highly correlated with propulsion and braking during walking [55–57]. Each IMU was mounted such that one axis moves along the sagittal plane. Specifically, the pelvis IMU was placed below the fifth lumbar vertebrae on the sacrum and the thigh and shank IMUs were placed laterally, a third of the corresponding segment length away from the knee and ankle joints respectively. For the purposes of this study, two additional IMUs were attached to the contralateral thigh and shank to enable indirect measurement of each limb’s AP-GRF concurrently. The calibration routines and algorithm were implemented in the same way for both the healthy and post-stroke subgroups, with indirect measurement of each limb’s AP-GRF estimated using measurements of global acceleration from the pelvis IMU and segment angles from the respective limb’s thigh and shank segment IMUs. That is, using this approach, only three IMUs (i.e., pelvis, shank, and thigh) are necessary for AP-GRF estimates for one limb, whereas five IMUs (1 pelvis, 2x shank, and 2x thigh) are necessary for AP-GRF estimates from both limbs.

The IMU measurements of pelvis global acceleration and thigh and shank angles (Fig. 2b) were used to generate subject-specific estimates of the AP-GRF time series (Fig. 2c), from which key metrics were extracted (see “Analyses” section). Both IMU and forceplate signals were temporally synchronized using a synchronization pulse triggered at the start of data collection in both data collection systems [58]. Whereas our IMU approach provided indirect measurements of the AP-GRF for all strides (Fig. 2c), only strides with full direct measurement of the individual limb AP-GRF by the forceplates (i.e., no partial strikes) were used for comparative analyses. Because the six forceplates were located within the middle 4m of one of the 10m straightaways of the walking track, steps taken during turns were not included in these analyses. For eight of the healthy study participants, data from the right limb are reported. For the remaining two healthy study participants, physical drift of one of the IMUs on the body during the experiment (due to an insecure attachment) made the right limb data unusable. Thus, for these two individuals, data from the left limb are reported. For the post-stroke participants, data from both the paretic and non-paretic limbs are reported. The model training datasets consisted of an average 14±3 strides per healthy participant and 9±4 strides per post-stroke participant (Supplementary Table 1).

### Data processing

IMU data were collected at 100Hz, with acceleration data filtered at 10Hz using a second order Butterworth filter. Forceplate data were collected at 2000Hz, filtered at 10 Hz using a second order Butterworth filter, and down sampled to match the IMU collection frequency. All gait data were segmented between initial contact and toe-off and time-normalized to 100 points to represent the stance phase of walking. When forceplate data were available, initial contact and toe-off events were defined using the vertical ground reaction force using a 30 N threshold. When forceplate data were not available, the maximum and minimum peaks in the IMU-measured shank angle were used, with initial contact defined by the maximum peak and toe-off defined by the minimum peak [43] (Fig. 2b).

Before modeling, the forceplate data were normalized to the body weight of the subject. The IMU orientations during the static standing trial served as the zero reference for the IMU orientation signals during walking [59, 60]. To compute the segment Euler angles, the quaternion vector relative to the static standing reference was found and subsequently rotated such that the roll axis of the IMU was aligned with the sagittal rotation axis of the segment [61–63]. The thigh and shank angles were offset to start at zero degrees at each periodic heel strike to address any drift over time [59, 64]. We also bounded the thigh and shank angles to a sine function, as is common with inverse kinematics equations [65]. Together, the filtered pelvis acceleration signal and the sine of the thigh and shank angles form the basis for the modeling approach described.

### Analyses

We analyzed the IMU data with two main goals. The first goal was to identify the IMU sensor set that provides the most accurate and reliable model-informed indirect measurement of the anterior-posterior ground reaction force (AP-GRF) time series. The second goal was to use this indirect measurement of the AP-GRF time series to estimate salient point metrics—i.e., the peaks, peak timings, and impulses of the anterior (propulsion) and posterior (braking) ground reaction force (see Fig. 1). A 75%-25% data split was used for model training and validation, respectively.

#### Indirect measurement of the anterior-posterior ground reaction force time series

**Fig. 3:**
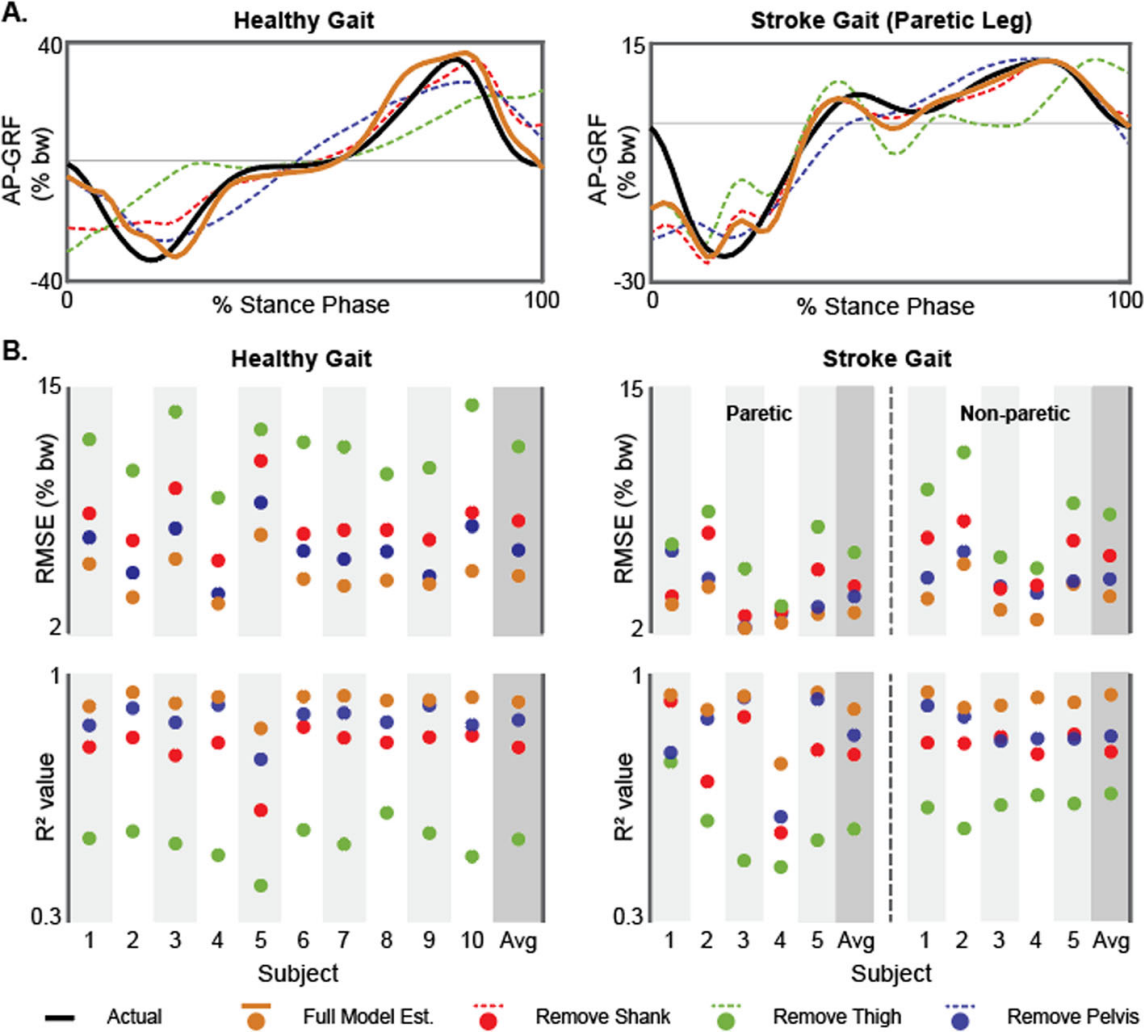
Comparison of direct (i.e., forceplate-measured) and indirect (i.e., IMU-based) measurements of the anterior-posterior ground reaction force (AP-GRF). **a** The AP-GRF time series reconstruction enabled by the primary sensor set (i.e., pelvis, thigh, and shank IMUs) and with each of these IMUs removed is shown for exemplar participants from the healthy and post-stroke cohorts. **b** Root mean square error (RMSE) and consistency (R^2^) metrics for each sensor set are shown for each study subject

Indirect measurements of the AP-GRF time series *F*_*a*−*p*,*est*_ were made by generating a subject-specific linear regression model using the IMU measurements of pelvis acceleration *a*_*pelvis*_, the sine of the thigh angle in the sagittal plane sin(*θ*_*thigh*_), the sine of the shank angle in the sagittal plane sin(*θ*_*shank*_), and the interactions sin(*θ*_*shank*_)×*a*_*pelvis*_, sin(*θ*_*thigh*_)×*a*_*pelvis*_, and sin(*θ*_*thigh*_)× sin(*θ*_*shank*_). Thus, we describe *F*_*a*−*p*,*est*_ as

1 $$ F_{a-p,est} = c \cdot x   $$

where *c* is a vector [*c*_1_...*c*_7_] of subject-specific regression coefficients generated from the training dataset and *x* is a vector [*a*_*pelvis*_, sin(*θ*_*thigh*_), sin(*θ*_*shank*_), sin(*θ*_*shank*_)×*a*_*pelvis*_, sin(*θ*_*thigh*_)×*a*_*pelvis*_, sin(*θ*_*thigh*_)× sin(*θ*_*shank*_)] of IMU measured components. To evaluate the relative importance of each IMU, we recomputed *F*_*a*−*p*,*est*_ without each of the pelvis, thigh, and shank IMUs—i.e., by setting their respective components in Eq. 1 to zero. The overall fit (*R*^2^) and the root mean square error (RMSE) when comparing *F*_*a*−*p*,*est*_ from each model to the AP-GRF time series directly measured by the forceplates (i.e., *F*_*a*−*p*,*act*_) is presented in Fig. 3.

#### Indirect measurement of propulsion and braking point metrics

Estimates of the propulsion and braking peak magnitudes were defined initially as the maximum and minimum values of the *F*_*a*−*p*,*est*_ time series. If more than one peak was observed, the second peak was used. To improve the accuracy of these point estimates, we introduced an additional subject-specific model that leveraged highly consistent estimates of the peak propulsion and braking timings, which were recorded as a function of the stance phase (%sp) and described as *F*_*pk*−*tim*,*est*_. More specifically, the values of *a*_*pelvis*_, sin(*θ*_*thigh*_), and sin(*θ*_*shank*_) at *F*_*pk*−*tim*,*est*_ were identified from the *F*_*a*−*p*,*est*_ time series and used to create a new vector $y = [1, a_{pelvis\_pk-tim}, \sin (\theta _{thigh\_pk-tim}), \sin (\theta _{shank\_pk-tim})]$, which was, in turn, used to describe *F*_*pk*−*mag*,*est*_ as

2 $$ F_{pk-mag,est} = d \cdot y   $$

where *d* is a vector [ *d*_1_... *d*_4_] consisting of updated regression coefficients. The interaction terms sin(*θ*_*shank*_)×*a*_*pelvis*_, sin(*θ*_*thigh*_)×*a*_*pelvis*_, and sin(*θ*_*thigh*_)× sin(*θ*_*shank*_) were not included in Eq. 2 as they did not improve the model’s performance.

In preliminary work, we found that *F*_*pk*−*tim*,*est*_ consistently overestimated or underestimated the actual force-plate measured peak timings and was thus revised as *F*_*pk*−*tim*,*revised*_ and described as

3 $$ F_{revised} = e_{1}+ e_{2} \cdot F_{est}   $$

The final point metrics of interest were the propulsion and braking impulses (*F*_*imp*,*est*_). These were computed by summing all of the positive (i.e., for propulsion) and negative (i.e., for braking) values in each *F*_*a*−*p*,*est*_ cycle and dividing by the total number of points (i.e., 100) to yield the propulsion and braking impulses (%bw) per stride. Similar to the peak propulsion and braking timings, we found that *F*_*imp*,*est*_ consistently overestimated or underestimated the actual propulsion and braking impulses and was thus revised using Eq. 3.

### Statistical analysis

All analyses were performed using custom MATLAB scripts (MATLAB, MathWorks, Natick, MA, USA). The regression model was fit using the fitlm() function in MATLAB, with *R*^2^ and root mean square error (RMSE) exported directly as a function output. Together, *R*^2^ and RMSE show the consistency and accuracy between the *F*_*a*−*p*,*act*_ and *F*_*a*−*p*,*est*_ time series.

For the point metrics of interest, in addition to computing the RMSE, the degree of absolute agreement among direct and indirect measurements was evaluated using two-way mixed effect, absolute agreement, single rater intraclass correlation coefficients (ICCs) [66, 68], with an alpha value of 0.05. ICC values were interpreted using the guidelines provided in [66] with an ICC above 0.90 considered to be excellent, 0.90 to 0.75 as good, 0.75 to 0.50 as moderate, and less than 0.50 as poor.

RMSEs and ICCs between direct and indirect measurements were computed for the training and validation datasets across all strides available from the healthy and post-stroke cohorts. Datapoints were identified as outliers if they were greater than three standard deviations from the mean. If an outlier was identified, the analysis was re-run with the datapoint removed. The results with and without the outlier are reported, and the outlier is shown in the plots.

## Results

### Measurement of the AP-GRF time series

The indirect measurement of the AP-GRF time series (*F*_*a*−*p*,*est*_) strongly approximated the direct measurement of the time series made by the reference standard forceplates (*F*_*a*−*p*,*act*_) in both the healthy (*R*^2^= 0.93, RMSE = 4.62%bw) and post-stroke (*R*^2^= 0.90, RMSE = 2.64%bw) cohorts. To assess the importance of each IMU used in the equation (Eq. 1), we recomputed *R*^2^ and RMSE with the removal of each IMU’s parameters (Fig. 3, Supplementary Table 1).

Removal of the thigh IMU parameters from Eq. 1 resulted in a substantial weakening of the AP-GRF reconstruction in both the healthy (*ΔR*^2^ = -0.40, % increase in RMSE = 155%) and post-stroke (*ΔR*^2^ = -0.34, % increase in RMSE = 125%) cohorts. Without the shank IMU parameters, the AP-GRF reconstruction was similarly weakened, but to a lesser degree, in both the healthy (*ΔR*^2^ = -0.13, % increase in RMSE = 66%) and post-stroke (*ΔR*^2^ = -0.13, % increase in RMSE = 55%) cohorts. Removal of the pelvis IMU parameters similarly weakened the AP-GRF reconstruction in both the healthy (*ΔR*^2^ = -0.06, % increase in RMSE = 31%) and post-stroke (*ΔR*^2^ = -0.07, % increase in RMSE = 33%) cohorts. Each IMU’s importance to the AP-GRF reconstruction varied across study participants (Fig. 3b, Supplementary Table 1).

### Measurement of AP-GRF point metrics

#### Peak propulsion magnitude

**Fig. 4:**
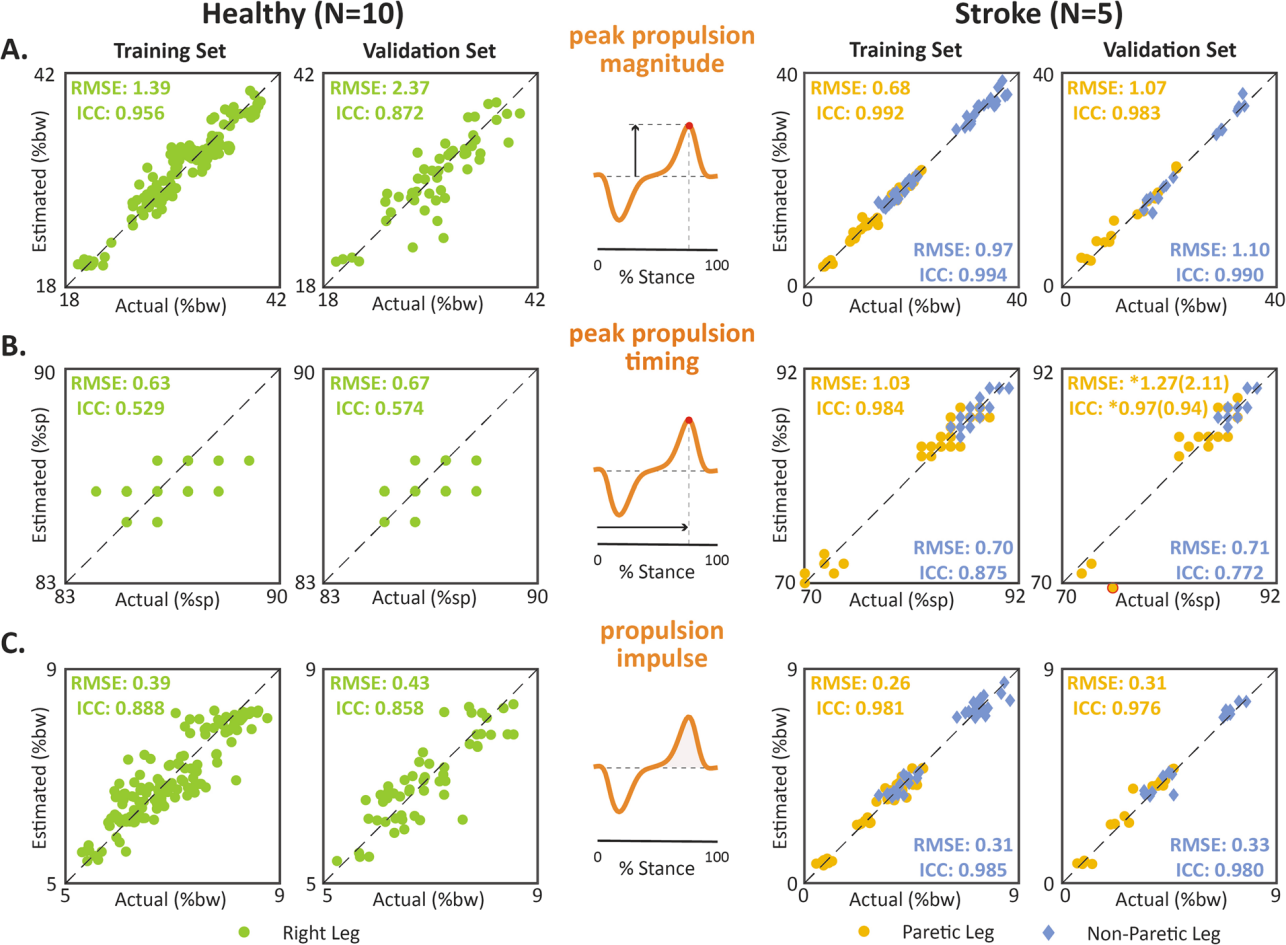
Accuracy of propulsion point metrics. Comparison of direct (i.e., forceplate) and indirect (i.e., IMU) measurements of the **a** peak propulsion magnitude, **b** peak propulsion timing, and **c** propulsion impulse. RMSE and ICC means are reported for each training and validation analysis. A single statistical outlier—identified with a red outline—was identified for the post-stroke peak propulsion validation analysis. The findings with and without the outlier are presented, respectively, in parentheses and with an *. Note: high homogeneity in **(b, Left)**, with multiple points overlaid. See Supplementary Table 2 for ICC 95% confidence interval

In the training dataset, the magnitude of the propulsion peaks in the *F*_*a*−*p*,*est*_ time series strongly approximated the *F*_*a*−*p*,*act*_ time series in the healthy cohort (RMSE = 1.39%bw, ICC = 0.96) and for both the paretic (RMSE = 0.68%bw, ICC = 0.99) and non-paretic (RMSE = 0.97%bw, ICC = 0.99) limbs of the post-stroke cohort (Fig. 4a, training). In the validation dataset, the estimated propulsion peak magnitudes remained a strong approximation of the forceplate-measured propulsion peak magnitudes for the healthy cohort (RMSE = 2.37%bw, ICC = 0.87) and for the paretic (RMSE = 1.07%bw, ICC = 0.98) and non-paretic (RMSE = 1.10%bw, ICC = 0.99) limbs of the post-stroke cohort (Fig. 4a, validation).

#### Peak propulsion timing

In the healthy cohort, the propulsion peak timings in the *F*_*a*−*p*,*est*_ time series strongly approximated the *F*_*a*−*p*,*act*_ time series for both the training (RMSE = 0.63%sp) and validation (RMSE = 0.67%sp) datasets (Fig. 4b). In the post-stroke cohort, similarly strong approximations were observed for both the paretic and non-paretic limb measurements in the training (paretic RMSE = 1.03%sp, non-paretic RMSE = 0.70%sp) and validation (paretic RMSE = 1.27%sp, non-paretic RMSE = 0.71%sp) datasets. High reliability was observed in the post-stroke training and validation datasets (ICCs >0.97); however, in the healthy datasets, highly homogeneous propulsion peak timings (i.e., between 84 to 89%sp) contributed to low reliability (ICCs <0.57), despite high agreement (see [66, 67]).

#### Propulsion impulse

In the training dataset, the propulsion impulses in the *F*_*a*−*p*,*est*_ time series strongly approximated those of the *F*_*a*−*p*,*act*_ time series in the healthy cohort (RMSE = 0.39%bw, ICC = 0.89) and for both the paretic (RMSE = 0.26%bw, ICC = 0.98) and non-paretic (RMSE = 0.31%bw, ICC = 0.99) limbs of the post-stroke cohort (Fig. 4c, training). In the validation dataset, the estimated propulsion impulses remained a strong approximation of the forceplate-measured propulsion impulses for the healthy cohort (RMSE = 0.43%bw, ICC = 0.86) and for the paretic (RMSE = 0.31%bw, ICC = 0.98) and non-paretic (RMSE = 0.33%bw, ICC = 0.98) limbs of the post-stroke cohort (Fig. 4c, validation).

#### Peak braking magnitude

**Fig. 5:**
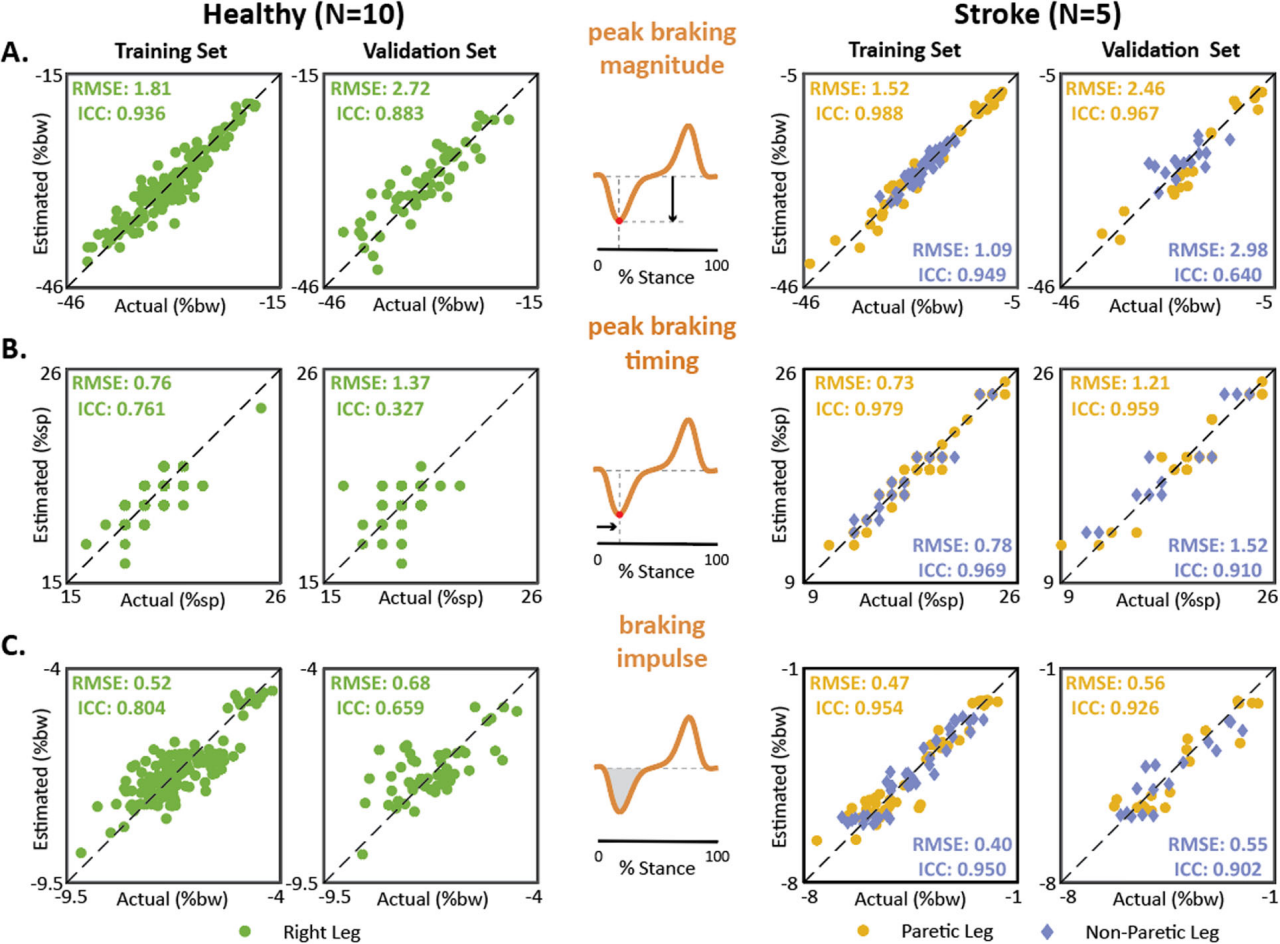
Accuracy of braking point metrics. Comparison of direct (i.e., forceplate) and indirect (i.e., IMU) measurements of the **a** peak braking magnitude, **b** peak braking timing, and **c** braking impulse. RMSE and ICC means are reported for each training and validation analysis. Note: high homogeneity in **(b, Left)**, with multiple points overlaid. See Supplementary Table 2 for ICC 95% confidence interval

In the training dataset, the magnitude of the braking peaks in the *F*_*a*−*p*,*est*_ time series strongly approximated the *F*_*a*−*p*,*act*_ time series in the healthy cohort (RMSE = 1.81%bw, ICC = 0.94) and for both the paretic (RMSE = 1.52%bw, ICC = 0.99) and non-paretic (RMSE = 1.09%bw, ICC = 0.95) limbs of the post-stroke cohort (Fig. 5a, training). In the validation dataset, the estimated braking peak magnitudes remained a strong approximation of the forceplate-measured braking peak magnitudes for the healthy cohort (RMSE = 2.72%bw, ICC = 0.88) and for the paretic (RMSE = 2.46%bw, ICC = 0.97) limbs of the post-stroke cohort (Fig. 5a, validation). The estimated braking peak magnitudes for the non-paretic limbs of the post-stroke cohort had a moderate approximation (RMSE = 2.98%bw, ICC = 0.64).

#### Peak braking timing

In the healthy cohort, the braking peak timings in the *F*_*a*−*p*,*est*_ time series strongly approximated the *F*_*a*−*p*,*act*_ time series for both the training (RMSE = 0.76%sp) and validation (RMSE = 1.37%sp) datasets (Fig. 5b). In the post-stroke cohort, similarly strong approximations were observed for both the paretic and non-paretic limb measurements made in the training (paretic RMSE = 0.73%sp, non-paretic RMSE = 0.78%sp) and validation (paretic RMSE = 1.21%sp, non-paretic RMSE = 1.52%sp) datasets. High reliability was observed in the post-stroke training and validation datasets (ICCs >0.96); however, in the healthy datasets, high homogeneity in the braking peak timings (i.e., between 16 to 22%sp) contributed to lower reliability (ICCs < 0.76), despite high agreement (see [66, 67]).

#### Braking impulse

In the training dataset, the braking impulses in the *F*_*a*−*p*,*est*_ time series strongly approximated those of the *F*_*a*−*p*,*act*_ time series in the healthy cohort (RMSE = 0.52%bw, ICC = 0.80) and for both the paretic (RMSE = 0.47%bw, ICC = 0.95) and non-paretic (RMSE = 0.40%bw, ICC = 0.95) limbs of the post-stroke cohort (Fig. 5c, training). In the validation dataset, the estimated braking impulses remained a strong approximation of the forceplate-measured braking impulses for the healthy cohort (RMSE = 0.68%bw, ICC = 0.66) and for the paretic (RMSE = 0.56%bw, ICC = 0.93) and non-paretic (RMSE = 0.55%bw, ICC = 0.90) limbs of the post-stroke cohort (Fig. 5c, validation).

## Discussion

Laboratory-based instrumented treadmills and forceplates are the gold standard in the direct measurement of the AP-GRF generated during walking [65]. We present the use of a minimal set of wearable inertial sensors to provide, with a high level of accuracy and robustness, indirect measurements of the AP-GRF time series and salient propulsion and braking point metrics. This work advances the development of point-of-care measurement systems that can catalyze the routine assessment and management of propulsion and braking locomotor deficits. Indeed, the approach presented in this paper has near-term potential to overcome logistical hinderances to including overground AP-GRF assessments in laboratory-based movement research and has long-term potential to extend the measurement capabilities of instrumented treadmills and forceplates to clinical and real-world settings.

Despite reduced ecological validity, instrumented treadmills are often utilized instead of overground forceplates in laboratory-based movement research [41, 69–73]. Indeed, instrumented treadmills facilitate the collection of a large number of consecutive samples with ease, whereas forceplate walkways have traditionally been limited to a small collection footprint of only several meters and require multiple trials to collect a small number of samples [65, 69]. Our sensor-based approach has the potential to extend the confined and limited length of forceplate walkways by providing indirect AP-GRF measurements for steps where a forceplate is not available, to replace partial forceplate strikes that are not usable, and to minimize threats to the validity of AP-GRF measurements that may arise when test subjects target the forceplates while walking (i.e., by only including steps without a forceplate in the analyses).

### Toward a minimal sensor set for AP-GRF time series measurements

When compared to forceplate measurements, three IMUs strategically mounted to the pelvis, thigh, and shank were effective in producing highly accurate indirect measurements of the AP-GRF generated during overground walking by both healthy and post-stroke individuals (Fig. 3). The selection of this sensor set was based on a biomechanical framework linking the AP-GRF generated during walking with certain kinematic features that are readily measured by IMUs. That is, the angles measured by the thigh and shank IMUs were used as a proxy for the orientation of the limb relative to the body and the acceleration measured by the pelvis IMU was used as proxy for the body’s center of mass acceleration. These variables are highly related to propulsion and braking function [44, 55–57] and our findings demonstrate the efficacy of this approach for reconstructing the AP-GRF time series.

We found that all three IMUs were required to produce the best estimates of the AP-GRF time series. Removing even one IMU had a substantial impact on estimation accuracy. Removal of the thigh IMU resulted in the greatest change across subjects: a 155% increase in the root mean square error and a 40% reduction in the model strength (*R*^2^) for healthy study participants, with a similar effect in the post-stroke cohort. Removing the pelvis or shank IMUs resulted in overall less reductions in accuracy; however, the effects varied across individual subjects (Fig. 3), indicating that including all three IMUs was most advantageous.

In recent work, a model-based approach to estimating the AP-GRF time series and other kinematic and kinetic variables during healthy walking used a 17-IMU sensor set to produce AP-GRF estimates with a root mean square error of 5.5%bw [48]. Our approach with only three IMUs resulted in lower error in healthy study participants (i.e., 4.6%bw) and was also shown to be highly effective for post-stroke hemiparetic gait, with error of 2.64%bw. Taken together with the availability and low cost of IMUs, these findings motivate future applications in both laboratory-based movement research and clinical practice—i.e., to complement and extend overground forceplate data collections in a research lab setting by providing indirect estimates of the AP-GRF time series every step, and providing clinicians with the access to these measurements that they need to inform locomotor diagnoses and treatments.

### Accuracy of AP-GRF point metric measurements

Point metrics are often extracted from the AP-GRF time series to characterize deficits in locomotor function [55, 74, 75]. The minimal detectable change (MDC) for many of these point metrics has been computed for hemiparetic walking overground [75]. Comparing the magnitude of error in our estimates to the available overground MDCs is another approach to evaluate measurement accuracy. For the peak paretic propulsion estimates, the root mean square error of 1.07%bw that we observed was lower than the 1.80%bw MDC previously reported [75]. Similarly, for the propulsion impulse estimates, the root mean square error of 0.31%bw for the paretic propulsion impulse was lower than the 0.90%bw MDC previously reported [75]. For the non-paretic limb, the propulsion peak and impulse point estimates had similar error magnitudes as the paretic limb, but MDC references were not available for comparison. The paretic limb’s braking point metrics were observed to have slightly higher error than the propulsion point metrics; however, MDC references were also not available for comparison. Ultimately, the accuracy of our approach is highlighted by the excellent agreement (ICCs >0.90) between IMU and forceplate measurements for most of the point metrics of interest.

In addition to the peak and impulse point metrics, we were also interested in the accuracy of the timing of the propulsion and braking peaks. Across subjects, the root mean square error in the timing of the propulsion and braking peaks was less than 1.4%sp. Despite this low error, the ICC values for the propulsion and braking peak timings were poor to moderate in the healthy cohort (ICCs ranged from 0.33 to 0.57), whereas they were good to excellent in the post-stroke cohort (ICCs ranged from 0.79 to 0.97). The low ICCs for the peak timings observed in the healthy cohort is likely the result of reduced variability in the dataset [66, 67]. In the healthy cohort, peak propulsion timings ranged between 84 and 89%sp and peak braking timings ranged between 16 and 22%sp, whereas substantially more variability was observed for both point metrics in the post-stroke cohort (Figs. 4 and 5b, Supplementary Table 1).

### Future considerations

An advantage of modeling the AP-GRF using a biomechanics-based equation is the potential to identify the relative contribution of each term, and how this relative contribution may change over time or as the result of intervention. Each of the terms included in the model reflects biomechanical processes related to the generation of the AP-GRF (i.e., segment/limb orientations and body acceleration) and examining changes in the relative contribution of each term—e.g., changes in standardized coefficients—would presumably reflect changes in these biomechanical processes. In contrast, model-free approaches centered on machine learning may provide more accurate estimates of the AP-GRF, but do not easily allow for examination of underlying biomechanical processes [49, 52]. Hybrid approaches that combine model and model-free terms are worth investigating. Furthermore, the extension of this work to other diagnostic groups with neuromotor impairments that alter the generation of anterior-posterior ground reaction forces (e.g., Multiple Sclerosis [5], Parkinson’s disease [25, 76], spinal cord injury [10], traumatic brain injury [11, 12]) would advance targeted rehabilitation approaches for these populations.

### Limitations

There are inherent limitations to using IMU technology for human movement analysis. For example, in addition to drift in the component signals that must be accounted for, IMUs need to be securely mounted to the body segments to avoid physical drift or movement of the sensor relative to the body segment and minimize soft tissue artifacts. As described in the “Methods” section, data from the right limbs of two healthy individuals were not usable due to an insecure attachment of the sensor to the segment. Relatedly, a current requirement of our IMU-based approach is mounting the lower limb IMUs such that one of the IMU planes correspond with the sagittal plane of the segment. Failure to do so would result in erroneous measurements of the sagittal plane angles used in the equations. Future efforts that leverage techniques such as coordinate rotations [60, 62, 63, 77] may enable extension of this approach. Fundamentally, our approach depends on body and limb kinematics to produce a kinetic measurement. Thus, patient subsets and walking conditions characterized by higher locomotor variability or AP-GRFs outside of the range observed in this study (e.g., acute post-stoke patients) may not be suitable for this approach in its current form.

### Conclusions

We show that indirect measurements of the anterior-posterior ground reaction forces generated during healthy and post-stroke walking can be made using three strategically-mounted inertial sensors in combination with subject-specific calibrations. This work has near-term potential to overcome logistical hinderances to including overground anterior-posterior ground reaction force assessments during laboratory-based movement research, and has long-term potential to catalyze the routine assessment and management of propulsion and braking deficits during locomotor rehabilitation. This foundational study is a step towards a fully wearable and autonomous point-of-care measurement system that can extend the measurement capabilities of laboratory-based instrumented treadmills and forceplates to ecological settings.

## Supplementary information

**Additional file 1** Supplementary Table 1: Changes in R^2^ and RMSE with removal of single IMUs. Supplementary Table 2: ICC 95% confidence intervals for propulsion and braking point metrics.

## Data Availability

Data will be made available upon reasonable request.
