# Supplementary material for: Indirect measurement of anterior-posterior ground reaction forces using a minimal set of wearable inertial sensors: from healthy to hemiparetic walking
Source: J Neuroeng Rehabil. 2020 Jun 29;17:82. doi: 10.1186/s12984-020-00700-7 (PMC7322880; doi:10.1186/s12984-020-00700-7)
Supplement: Supplementary file 1 — Additional file 1 Supplementary Table 1: Changes in R2 and RMSE with removal of single IMUs. Supplementary Table 2: ICC 95% confidence intervals for propulsion and braking point metrics. [file 12984_2020_700_MOESM1_ESM.pdf]

Table 1 **Changes in the performance of the AP-GRF estimation model for each study participant when removing one of the IMUs used in the three-sensor set in equation 1**

participant	RMSE (%bw)				R^2				number
number	full model	remove shank	remove thigh	remove pelvis	full model	remove shank	remove thigh	remove pelvis	of step
**Healthy study participants**									
H01	5.291	8.067	12.182	6.740	0.912	0.796	0.535	0.858	13
H02	3.426	6.590	10.453	4.790	0.952	0.823	0.555	0.907	14
H03	5.559	9.462	13.727	7.245	0.921	0.772	0.520	0.866	13
H04	3.079	5.474	8.942	3.617	0.939	0.808	0.487	0.916	12
H05	6.889	10.999	13.734	8.673	0.849	0.615	0.400	0.761	19
H06	4.448	6.939	12.033	6.005	0.940	0.853	0.559	0.890	17
H07	4.063	7.156	11.767	5.548	0.942	0.822	0.518	0.893	9
H08	4.368	7.164	10.263	5.975	0.929	0.809	0.608	0.867	14
H09	4.170	6.626	10.603	4.596	0.930	0.824	0.550	0.915	12
H10	4.890	8.111	14.098	7.371	0.938	0.829	0.483	0.859	19
Avg±Std	4.62±1.10	7.66±1.59	11.78±1.72	6.05±1.51	0.93±0.03	0.80±0.07	0.53±0.06	0.87±0.05	14±3
**Post-stroke study participants (Paretic Limb)**									
S01	2.999	3.453	6.345	5.991	0.945	0.927	0.753	0.780	11
S02	3.972	6.972	8.151	4.434	0.902	0.697	0.585	0.877	15
S03	1.670	2.361	4.993	1.722	0.941	0.882	0.471	0.937	7
S04	1.979	2.642	2.917	2.508	0.748	0.551	0.453	0.596	9
S05	2.564	5.004	7.355	2.946	0.943	0.781	0.527	0.924	3
Avg±Std	2.64±0.91	4.09±1.91	5.95±2.07	3.52±1.70	0.90±0.08	0.77±0.15	0.56±0.12	0.82±0.14	9±4
**Post-stroke study participants (Non - Paretic Limb)**									
S01	3.323	6.699	9.389	4.481	0.953	0.808	0.623	0.914	6
S02	5.251	7.629	11.458	5.942	0.908	0.806	0.563	0.882	13
S03	2.697	3.872	5.637	4.003	0.915	0.825	0.630	0.813	8
S04	2.154	4.065	5.014	3.639	0.937	0.776	0.658	0.820	6
S05	4.216	6.602	8.644	4.371	0.867	0.673	0.440	0.857	6
Avg±Std	3.53±1.23	5.77±1.70	8.03±2.68	4.49±0.88	0.92±0.03	0.78±0.06	0.58±0.09	0.86±0.04	

Table 2 ICC 95% confidence interval for all reported point metrics

	Healthy		Post-stroke (paretic)		Post-stroke (non-paretic)	
	training set	validation set	training set	validation set	training set	validation set
peak A-GRF magnitude	[0.94, 0.97]	[0.79, 0.92]	[0.99, 1.00]	[0.96, 0.99]	[0.99, 1.00]	[0.97, 1.00]
peak A-GRF timing	[0.40, 0.63]	[0.36, 0.73]	[0.97, 0.99]	[0.93, 0.99]	[0.78, 0.93]	[0.45, 0.91]
A-GRF impulse	[0.85, 0.92]	[0.77, 0.92]	[0.96, 0.99]	[0.94, 0.99]	[0.97, 0.99]	[0.95, 0.99]
peak P-GRF magnitude	[0.91, 0.95]	[0.80, 0.93]	[0.98, 0.99]	[0.91, 0.99]	[0.90, 0.97]	[0.23, 0.86]
peak P-GRF timing	[0.68, 0.82]	[0.06, 0.54]	[0.96, 0.99]	[0.90, 0.98]	[0.94, 0.98]	[0.77, 0.97]
P-GRF impulse	[0.74, 0.86]	[0.48, 0.79]	[0.92, 0.97]	[0.82, 0.97]	[0.91, 0.97]	[0.75, 0.96]
